# Epstein-Barr Virus (EBV) Masquerading as Exudative Tonsillitis and Rash in a Transgender Adolescent

**DOI:** 10.7759/cureus.87737

**Published:** 2025-07-11

**Authors:** Amrita A Gujar, Kimberly Pernudi, Adebayo Adeyinka, Noah Kondamudi

**Affiliations:** 1 Pediatrics, The Brooklyn Hospital Center, Brooklyn, USA; 2 Pediatric Emergency Medicine, The Brooklyn Hospital Center, Brooklyn, USA

**Keywords:** allergy and anaphylaxis, amoxicillin-associated rash, atypical rash, ebv positive, transgender medicine

## Abstract

A 19-year-old transgender adolescent on hormonal therapy with a history of recent hospitalization for exudative tonsillitis presented with worsening sore throat, dysphagia, fever, and a diffuse, generalized macular rash involving the back, face, abdomen, arms, and legs. Despite prior antibiotic treatment with amoxicillin, her symptoms recurred, prompting further evaluation. Laboratory findings revealed leukocytosis with lymphocytic and monocytic predominance, elevated inflammatory markers, and a positive Epstein-Barr virus (EBV) test, confirming infectious mononucleosis. Imaging demonstrated persistent tonsillitis without abscess formation, and additional testing identified concurrent herpes simplex virus (HSV)-1 oral ulcers. Given persistent fevers and systemic inflammation, hemophagocytic lymphohistiocytosis (HLH) was considered but not confirmed. Management included IV clindamycin for tonsillitis, corticosteroids for airway inflammation, and doxycycline for atypical pneumonia. Over the 10-day hospitalization, the patient showed gradual improvement, with resolution of the rash and all other symptoms. The rash was ultimately diagnosed as an amoxicillin-induced rash associated with EBV infection. This case highlights the importance of recognizing amoxicillin-induced rash in EBV infection and distinguishing it from allergic reactions and other common rashes, including but not limited to scarlet fever, viral exanthem, drug reactions, and HLH. Although various skin rashes are common in the transgender population, a literature review found no evidence linking amoxicillin-induced EBV rash to transgender patients. Accurate diagnosis aids in the appropriate selection of antimicrobial therapy and helps avoid unnecessary antibiotic restrictions due to misattributed allergic reactions.

## Introduction

Infectious mononucleosis, caused by the Epstein-Barr virus (EBV), is a viral illness that typically presents with fever, pharyngitis, lymphadenopathy, and fatigue. While most cases follow a benign course, complications such as drug-induced hypersensitivity reactions can complicate diagnosis and management. An underrecognized phenomenon is the development of a diffuse erythematous rash in patients with EBV infection who are exposed to aminopenicillin antibiotics, such as amoxicillin [1-3]. This reaction can closely mimic EBV-associated exanthema, making it challenging to distinguish between a viral rash and a drug-induced hypersensitivity response [4-7].

Here, we present the case of a 19-year-old transgender male transitioning to female with a medical history of gender transition with hormone replacement therapy and a recent hospitalization for exudative tonsillitis, who was readmitted with worsening symptoms. Given the severity of her presentation, a broad differential diagnosis was considered, including scarlet fever, viral exanthem, drug reaction, and hemophagocytic lymphohistiocytosis (HLH) [8,9]. After thorough evaluation and workup, she was diagnosed with amoxicillin-induced rash associated with infectious mononucleosis and atypical pneumonia. This case highlights the diagnostic challenges in differentiating EBV-associated rash from amoxicillin-induced rash in EBV infection and underscores the importance of a detailed clinical history and careful evaluation [10].

## Case presentation

A 19-year-old transgender male transitioning to female, with a preference to be addressed as a female, admitted to the pediatric intensive care unit (PICU) one week ago for exudative tonsillitis, presented to the emergency department (ED) with a three-day exacerbation of her symptoms. She reported worsening sore throat and severe dysphagia, partially relieved by Tylenol. One week prior, she was treated for respiratory distress and suspected right peritonsillar abscess, improving after initial treatment with amoxicillin and sulbactam intravenously, and was discharged home with oral amoxicillin and clavulanic acid. She returned to the ED on day 4 of oral antibiotics with a new-onset rash and recurrence of her previous symptoms. 

**Figure 1:**
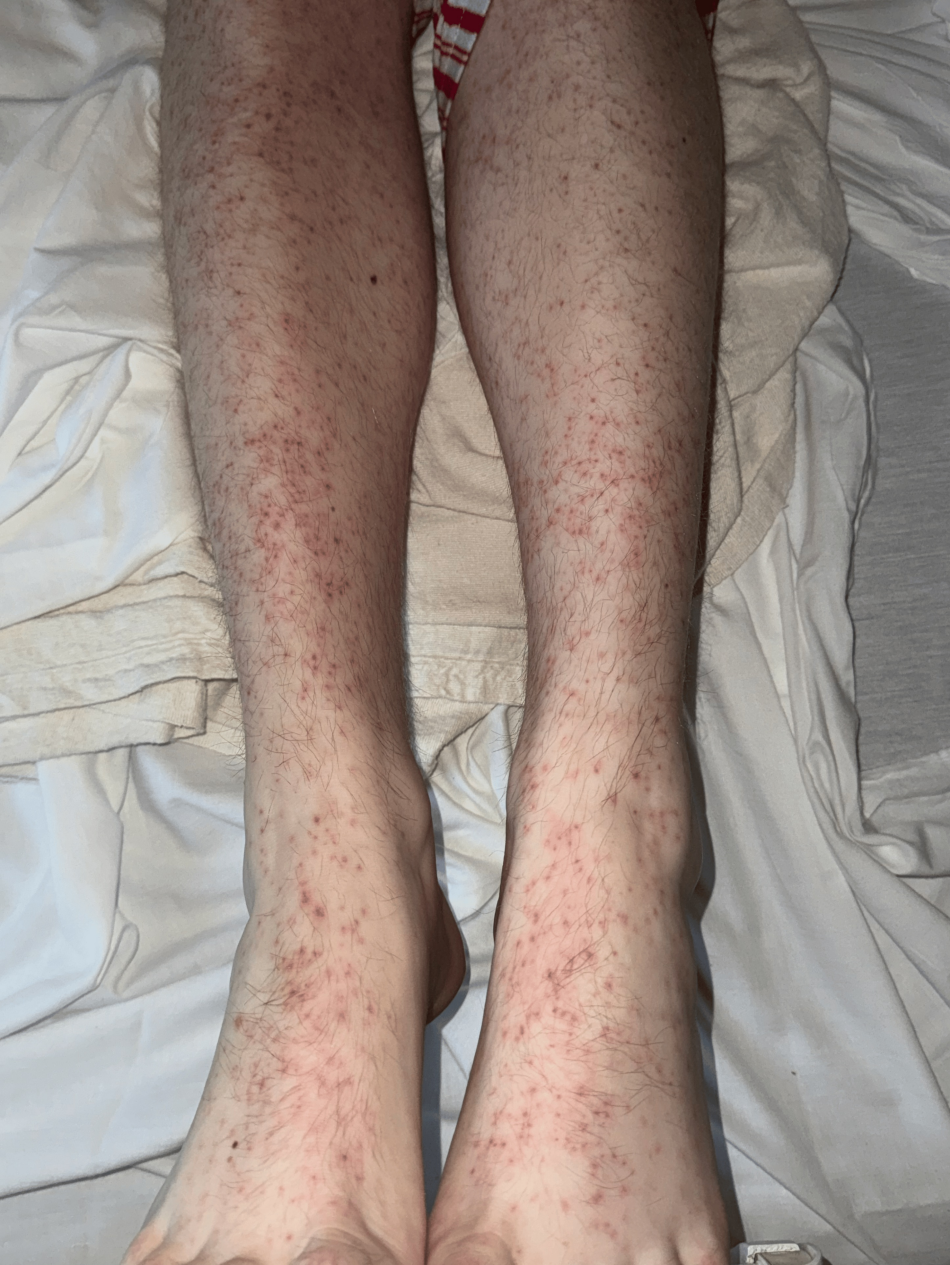
Erythematous, macular, blanching rash on the bilateral legs

**Figure 2:**
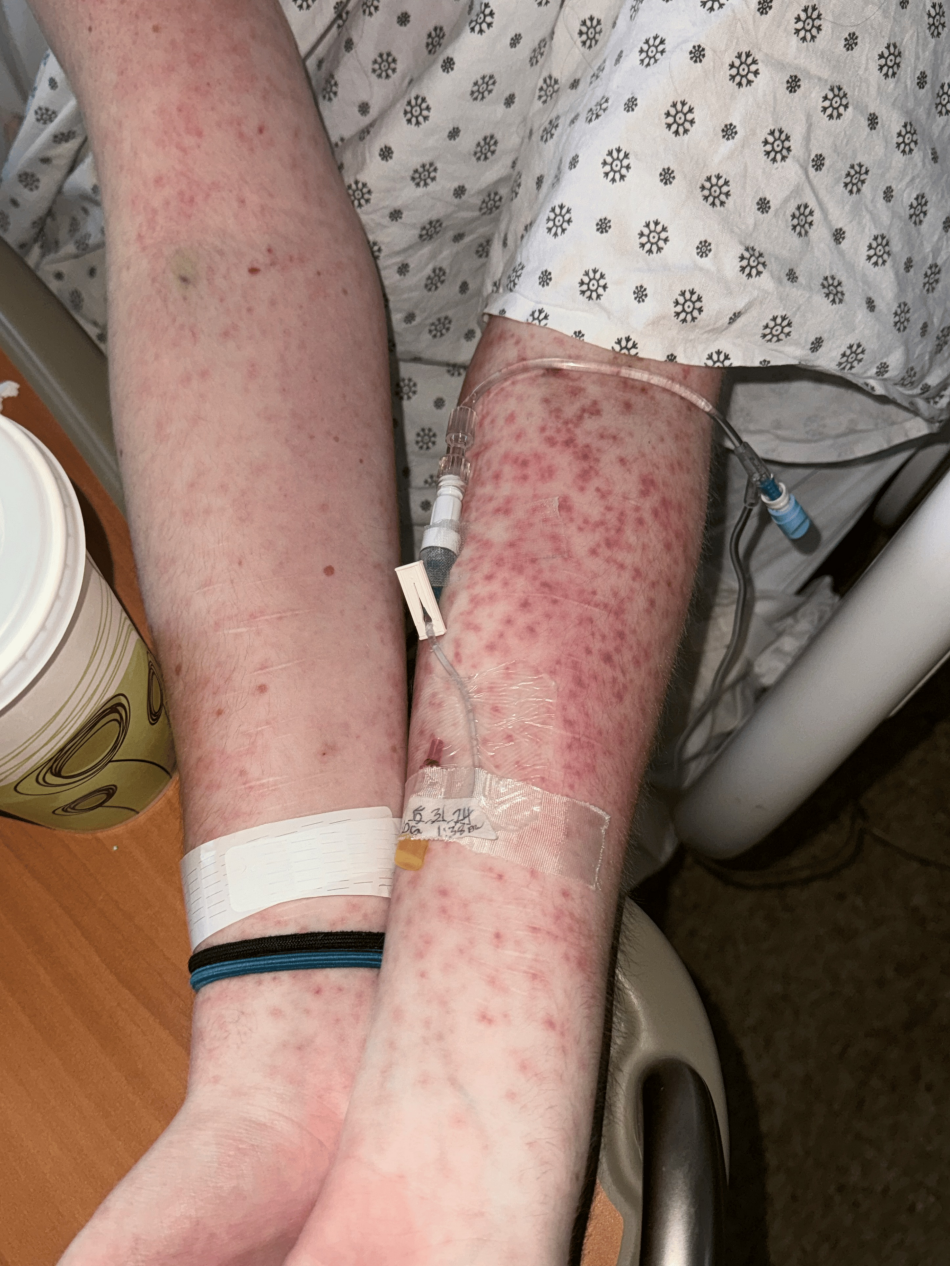
Erythematous, macular, blanching rash on the bilateral arms

**Figure 3:**
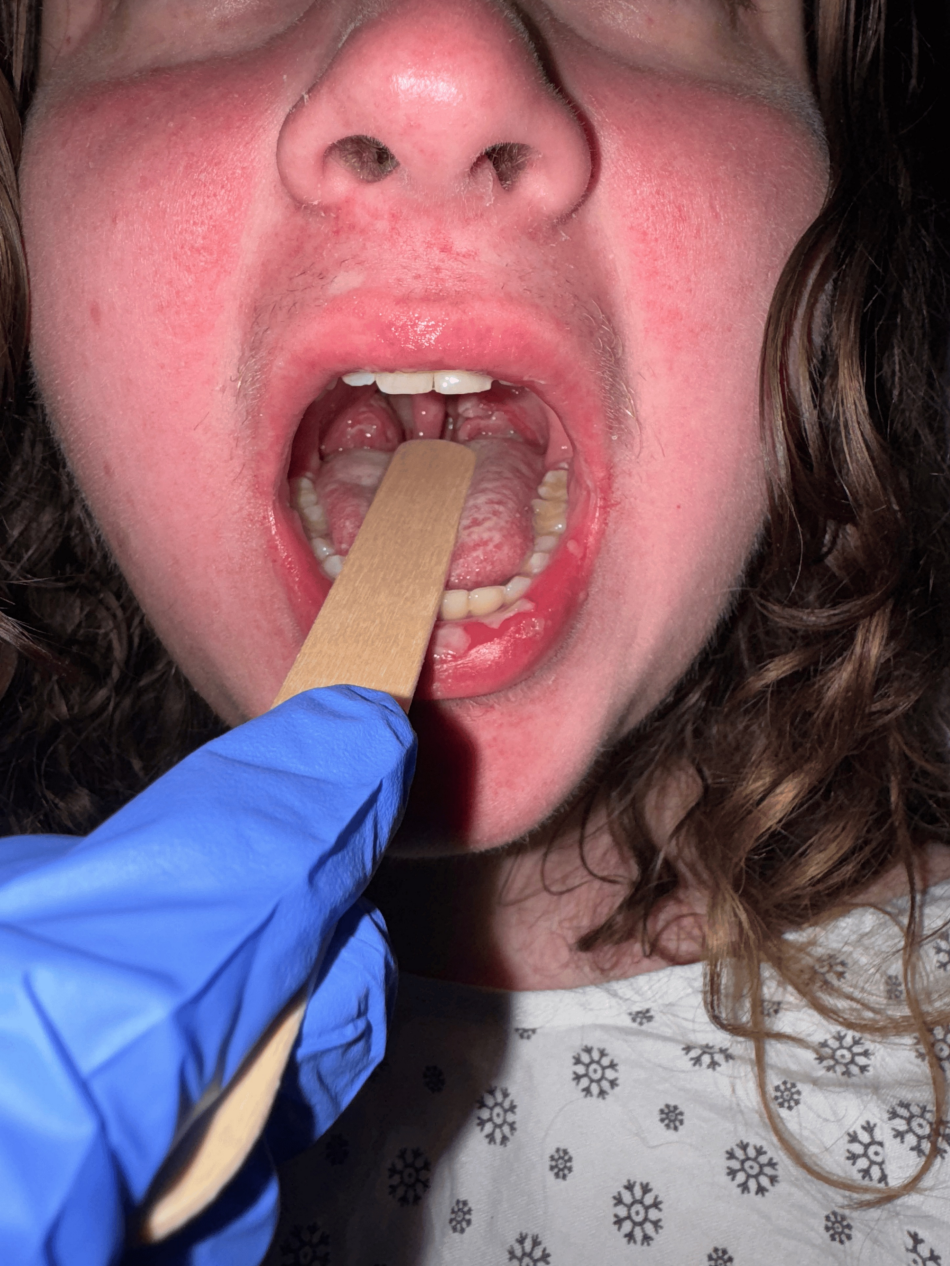
Tonsillitis with exudates and rash around the lips

Upon her second ED presentation, the patient appeared to be in distress and had open-mouthed, noisy breathing, preferring to sit up with neck extension. Her vital signs were remarkable for fever (101.7°F) and tachycardia (145 bpm). Notable findings on physical exam included an erythematous, macular, blanching rash on the bilateral arms, back, and torso (see Figures 1-3), capillary refill of less than two seconds, and bilateral grade IV enlarged erythematous tonsils with exudates. There were accumulated salivary secretions, small, white aphthous ulcers on the lower lip, dry and cracked lips, and halitosis. The patient had a muffled "hot potato" voice, along with fullness on the right side of the neck, accompanied by cervical lymphadenopathy. No other lymphadenopathy was noted, and the abdominal exam was unremarkable with a soft, non-tender abdomen with no organomegaly. No other vesicular rash was noted on the body. The patient reported being sexually active with only male partners and always using condoms, with the last sexual activity two months ago. A sexually transmitted disease (STD) panel was done (Table 1). Laboratory results showed an elevated C-reactive protein (56 mg/L), low CO2 (20 mmol/L), an anion gap of 15 mmol/L, and leukocytosis (white blood cell (WBC): 21×10^9^/L) with elevated lymphocytes (47.4%) and monocytes (11.1%). Blood culture showed no growth. A computed tomography (CT) scan of the neck revealed persistent, though reduced, palatine tonsil enlargement consistent with tonsillitis, stable cervical lymphadenopathy, and right upper lobe airspace opacities. She was admitted to the pediatric floor for hydration, pain management, and airway monitoring. 

**Table 1:** All lab tests with results and reference values done during the admission HIV: human immunodeficiency virus; HSV-1 PCR: herpes simplex virus type 1 polymerase chain reaction

Test	Result	Reference range
HIV viral load	Negative	Negative
Viral panel assay	Negative	Negative
Urinalysis (chlamydia, gonorrhea)	Negative	Negative
HSV-1 PCR (oral ulcers)	Positive	Negative
*Mycoplasma* IgM	Negative	Negative
Throat culture	No growth (final)	No growth (final)
Blood culture	No growth (final)	No growth (final)

Inpatient management commenced with IV clindamycin (600 mg every eight hours) for broad-spectrum coverage of the persistent tonsillitis and acetaminophen for pain management, and her home medications of spironolactone and estradiol were continued. Due to persistent fevers and a muffled voice, a repeat CT scan of the neck with contrast was performed on hospital day 4 to rule out abscess formation. This imaging re-demonstrated persistent tonsillitis/pharyngitis with phlegmonous changes, similar to the previous CT findings. Consequently, clindamycin was continued for 10 days, and dexamethasone (12 mg IV daily) was initiated for five days to address airway inflammation. The rash worsened over the course of admission. An EBV viral capsid antigen IgM antibodies (VCA Ab IgM) returned positive, confirming infectious mononucleosis. Given the persistent fever, worsening rash, lymphocytosis, and elevated inflammation markers, HLH was considered. However, the absence of critical HLH markers (e.g., significantly elevated ferritin, cytopenias, and hepatosplenomegaly) suggested that the inflammatory response was more consistent with severe EBV infection rather than HLH. EBV is a known trigger for HLH, so trending inflammatory markers was appropriate in this case (Table 2).

**Table 2:** Lab trends over the course of hospitalization WBC: white blood cell; AST: aspartate aminotransferase; ALT: alanine aminotransferase; CRP: C-reactive protein

Labs	5/21/24	5/27/24	5/28/24	5/30/24	6/2/24	Reference ranges
Hematology						
Hemoglobin (g/dl)	13.9	14.1	13.8	12.9	12.2	12-16
Platelets (10³/µL)	-	222	282	287	239	150-400
WBC (10³/µL)	17.6	21	13.4	14.9	12.9	4-11
Sedimentation rate (ml/hr)	-	-	-	38	21	0-20
General chem						
AST (U/L)	-	-	-	20	15	10-40
ALT (U/L)	-	-	-	36	25	7-46
Triglycerides (mg/dl)	-	-	-	128	198	<150
Ferritin (ng/ml)	-	-	-	803	644	20-300
CRP (mg/L)	53.87	55.6	-	-	10.5	<5

The patient's rash improved to a mild maculopapular form limited to her back, and her tonsillitis significantly improved after the discontinuation of amoxicillin. She tolerated oral fluids and solid foods two days before discharge. After an extended hospital stay for 10 days for persistent tonsillitis, infectious mononucleosis, and atypical pneumonia, the patient was discharged in stable condition with a final diagnosis of amoxicillin-induced rash in infectious mononucleosis. 

## Discussion

This case highlights the challenges of managing severe tonsillitis in the context of EBV infection, complicated by antibiotic-induced rash, airway inflammation, and atypical pneumonia. The patient, previously hospitalized for exudative tonsillitis, returned with worsening symptoms despite an ongoing course of Augmentin. Persistent fever, tachycardia, and lymphocytosis raised suspicion for EBV, later confirmed via testing. Imaging showed ongoing tonsillitis without abscess formation, suggesting a prolonged inflammatory response rather than bacterial superinfection. 

The incidence of rash after aminopenicillin treatment in children with EBV infection was reported to be 80-100%. In pediatric populations, a study by Chovel-Sella et al. found that the incidence of amoxicillin-associated EBV rash was only 29.5%, and Dibek Misirlioglu et al. reported a similar reduction, with an incidence of 16.6% in children treated with amoxicillin [1,2]. 

A study by Renn et al. demonstrated that young adults with EBV infection often develop a maculopapular exanthema after taking amoxicillin, with some cases showing evidence of drug-specific sensitization [3]. This suggests that while the rash remains a notable clinical phenomenon, it may not occur as universally as once assumed. Furthermore, the immune mechanisms behind the rash appear to be complex and are likely influenced by both the viral infection and the immune response to the antibiotic. 

Diagnosing amoxicillin-induced rashes in EBV-infected patients can be challenging due to the similarity between the rash caused by EBV itself and the rash induced by the antibiotic. The rash caused by EBV infection and the amoxicillin-induced rash in EBV-infected patients can be distinguished by timing, characteristics, and resolution. EBV rash typically appears early in the infection, is morbilliform, and resolves as the viral illness improves. In contrast, the amoxicillin-induced rash usually develops 3-10 days after starting the antibiotic, tends to be more intense and localized, and fades after amoxicillin is discontinued. This transient nature of the rash suggests that it may be due to a temporary immune alteration induced by the virus, rather than a true, long-lasting drug allergy. This underscores the importance of careful diagnosis and highlights the need for clinicians to avoid prematurely labeling patients as allergic to amoxicillin based solely on the presence of a rash during EBV infection. 

The pathogenesis of the rash in EBV-infected patients treated with amoxicillin is thought to be primarily linked to a transient immune alteration induced by the EBV infection itself, rather than a classic IgE-mediated allergic reaction. EBV infection is known to induce immune changes, such as increased activation of CD8+ T cells and decreased IL-10 production, which may predispose individuals to hypersensitivity reactions when amoxicillin is introduced into the system [4]. These immune changes could trigger a delayed-type IV hypersensitivity reaction, which is a hallmark of the amoxicillin rash seen in these patients. Unlike typical allergic reactions, which are usually immediate, the delayed nature of the rash in EBV patients suggests a more complex immunological mechanism at play. 

Studies have demonstrated drug-specific lymphocyte proliferation in EBV patients with amoxicillin-induced rashes, suggesting that T-cell activation is a crucial component of this immune response [3]. This is in contrast to typical allergic reactions, which usually involve IgE antibodies, and highlights that the rash seen in these patients is not a classic allergic response. Furthermore, some patients show evidence of true drug sensitization, as indicated by positive skin tests or in vitro lymphocyte transformation tests, suggesting that the sensitization may be persistent rather than transient [4]. This further complicates the understanding of amoxicillin-induced rashes in EBV patients, as it raises the possibility that some individuals may develop a long-term sensitivity to the drug. 

The clinical implications of these findings are significant. Since most amoxicillin-induced rashes in EBV patients are not true drug allergies, clinicians should refrain from labeling these patients as penicillin-allergic solely on the basis of a rash. Doing so could lead to unnecessary avoidance of amoxicillin and other beta-lactam antibiotics in the future, limiting treatment options for these patients. Accurate differentiation between a transient, EBV-related reaction and a true drug hypersensitivity reaction is crucial for selecting appropriate antibiotics in future infections (Table 3). Allergy testing may be necessary in some cases to confirm or rule out a true drug allergy, but clinicians should be aware that the presence of a rash during EBV infection is not always indicative of a true allergy [3,4]. Mislabelling as an allergy can lead to unnecessary lifelong avoidance of penicillins and other beta-lactam antibiotics [5-7].

**Table 3:** Differences between amoxicillin anaphylactic rash, regular EBV rash, and amoxicillin-induced EBV rash EBV: Epstein-Barr virus

Feature	Amoxicillin anaphylactic rash	Regular EBV rash	Amoxicillin-induced EBV rash
Cause	IgE-mediated allergic reaction (type I hypersensitivity)	EBV infection	Non-allergic, immune-mediated drug reaction in EBV-infected patients (type IV hypersensitivity)
Timing of onset	Minutes to hours after drug exposure	Typically 4-6 days after symptom onset	5-10 days after starting amoxicillin
Rash appearance	Raised, erythematous, well-demarcated wheals (hives), intensely pruritic; may coalesce	Diffuse, faint pink to red maculopapular rash; non-itchy; begins on trunk and spreads outward	Widespread, symmetric, erythematous maculopapular rash, often more prominent on the trunk and extremities; non-itchy
Progression	May evolve into angioedema or anaphylaxis with systemic symptoms	Gradually fades over a few days without treatment	Resolves gradually after stopping amoxicillin, typically within a week
Other symptoms	Wheezing, difficulty breathing, hypotension, gastrointestinal symptoms	Fever, severe pharyngitis, cervical lymphadenopathy, fatigue	Fever, pharyngitis, lymphadenopathy, fatigue
Resolution	Requires immediate treatment with epinephrine, antihistamines, and steroids	Resolves spontaneously as EBV infection improves	Resolves once the drug is discontinued; antihistamines or steroids usually not needed
Re-challenge with amoxicillin	Contraindicated due to the risk of a severe reaction	No contraindication	Rash will likely recur but is not a true allergic reaction

In male-to-female patients undergoing estrogen therapy and testosterone blockade, various dermatologic conditions have been observed, including melasma, lichen planus, systemic sclerosis, hormone-resistant hirsutism, pseudofolliculitis barbae, eczema, and xerosis. However, the rash observed in our patient did not align with any of these diagnoses [7-10]. A literature review did not identify any association between an increased incidence of amoxicillin-induced EBV rash and patients undergoing gender affirmation therapy. 

## Conclusions

Our case demonstrates that amoxicillin-induced rash, although uncommon, continues to occur in pediatric patients. Given the transient nature of the rash, it is critical for clinicians to avoid misdiagnosing this reaction as a true drug allergy. Mislabelling as an allergy can lead to unnecessary lifelong avoidance of penicillins and other beta-lactam antibiotics. It could restrict future antibiotic choices, potentially leading to suboptimal treatment options and increased antibiotic resistance due to reliance on broader-spectrum agents. It is unclear if there is an association between hormonal therapy and the occurrence of this reaction.

Since this reaction is not a true allergic response in most cases, clinicians should be cautious in diagnosing penicillin allergy and consider re-evaluation with allergy testing when appropriate. In select cases, drug hypersensitivity testing, such as skin testing or lymphocyte transformation tests, may be warranted to clarify the diagnosis and guide future antibiotic use. 
